# Mechanosensitive Piezo channels mediate the physiological and pathophysiological changes in the respiratory system

**DOI:** 10.1186/s12931-022-02122-6

**Published:** 2022-07-29

**Authors:** Huaiyu Xiong, Jing Yang, Jun Guo, Aijia Ma, Bo Wang, Yan Kang

**Affiliations:** Department of Critical Care Medicine, West China Hospital of Sichuan University, No. 17, Section 3, Renmin South Road, Wuhou District, Chengdu, 610000 Sichuan China

**Keywords:** Mechanosensitive, Piezo, Pathophysiological, Respiratory system

## Abstract

Mechanosensitive Piezo ion channels were first reported in 2010 in a mouse neuroblastoma cell line, opening up a new field for studying the composition and function of eukaryotic mechanically activated channels. During the past decade, Piezo ion channels were identified in many species, such as bacteria, *Drosophila*, and mammals. In mammals, basic life activities, such as the sense of touch, proprioception, hearing, vascular development, and blood pressure regulation, depend on the activation of Piezo ion channels. Cumulative evidence suggests that Piezo ion channels play a major role in lung vascular development and function and diseases like pneumonia, pulmonary hypertension, apnea, and other lung-related diseases. In this review, we focused on studies that reported specific functions of Piezos in tissues and emphasized the physiological and pathological effects of their absence or functional mutations on the respiratory system.

**Supplementary Information:**

The online version contains supplementary material available at 10.1186/s12931-022-02122-6.

## Introduction

Mechanotransduction is a fundamental physiological process that converts mechanical stimuli into bioelectrical and biochemical signals [1] and affects the cellular response to external changes and the growth and development of skeletal organs. Mechanical forces that stimulate cell membrane and intracellular signals rapidly activate mechanically activated (MA) ion channels [2]. Previous studies have identified ion channels in bacteria, moss [3], *Drosophila* [4], and mammals [5]. In bacteria, these channels respond to osmotic shock by permeating ions and osmolytes to prevent cell lysis [6]. In mammals, basic life activities, such as the sense of touch [7], proprioception [8], hearing [9], vascular development, and blood pressure regulation, depend on MA ion channels to initiate membrane depolarization in excitable cells and Ca^2+^ conduction in non-excitable cells [10–12]. Therefore, accurate mechanotransduction is essential for normal organ function, while abnormal or faulty mechanotransduction may lead to a variety of diseases.

The discovery of Piezos in 2010 opened up a new field for studying the composition and function of eukaryotic MA channels [10]. Piezo ion proteins have been identified as pore-forming subunits of non-selective cationic mechanosensitive ion channels, the expression of which is required for several processes involving mechanotransduction [13]. During the past decade, studies have indicated that Piezos are expressed in several tissues. Piezo1 ion channel is mainly expressed in non-excitable cells, such as endothelial cells (ECs) and adipocytes (Additional file 1: Figure S1). In contrast, the Piezo2 ion channel mainly exists in sensory neurons, such as dorsal root ganglia (DRG) [14], Merkel cells [15], skeletal tissues, bladder urethral epithelial cells [16], and enterochromaffin cells [17] (Additional file 1: Figure S2).

In addition, cumulative evidence suggests that Piezo ion channels play a major role in lung vascular development and function [18] and diseases like pneumonia [19], pulmonary hypertension (PH) [20], apnea [21], and other lung-related diseases. Changes in the mechanical properties and conditions of the airway and lungs, such as lung contraction and inflation, activate airway mechanical sensors [22]. Piezo, as a mechanosensitive ion protein, plays a crucial role in the pathophysiological response of the lung. Therefore, the current review aimed to briefly summarize the structure of Piezo ion channels and the diseases caused by deletion or mutation in Piezos in different tissues and organs, and then focus on discussing how Piezos might be involved in the physiology and pathophysiology of the respiratory system.

### Discovery of Piezo1 and Piezo2

Bertrand Coste et al. were the first to identify that mechanosensitive Piezo ion channels rapidly respond to mechanical stimuli and mediate the influx of cations into cells to induce cellular excitation and signal transduction [10]. Bertrand Coste et al. identified Fam38A by selecting known cation channels and proteins of unknown function from the mouse neuroblastoma Neuro2A cell line and knocking out the genes one by one [10]. Since Fam38A encodes a protein required for stress-activated ion channel expression, it was named Piezo1, which means "pressure" in Greek. Piezo2 was later identified as a gene homologous to Piezo1 [2]. According to the NCBI database, only one Piezo member exists in plants, invertebrates, and *Drosophila melanogaster* but two in vertebrates [23]. Piezo is the largest transmembrane ion channel identified so far containing 114 transmembrane domains (TMs). Moreover, they show no sequence homology with known ion channels [24]. This groundbreaking finding has attracted the attention of several researchers worldwide.

### Structure of Piezo1 and Piezo2

**Fig. 1:**
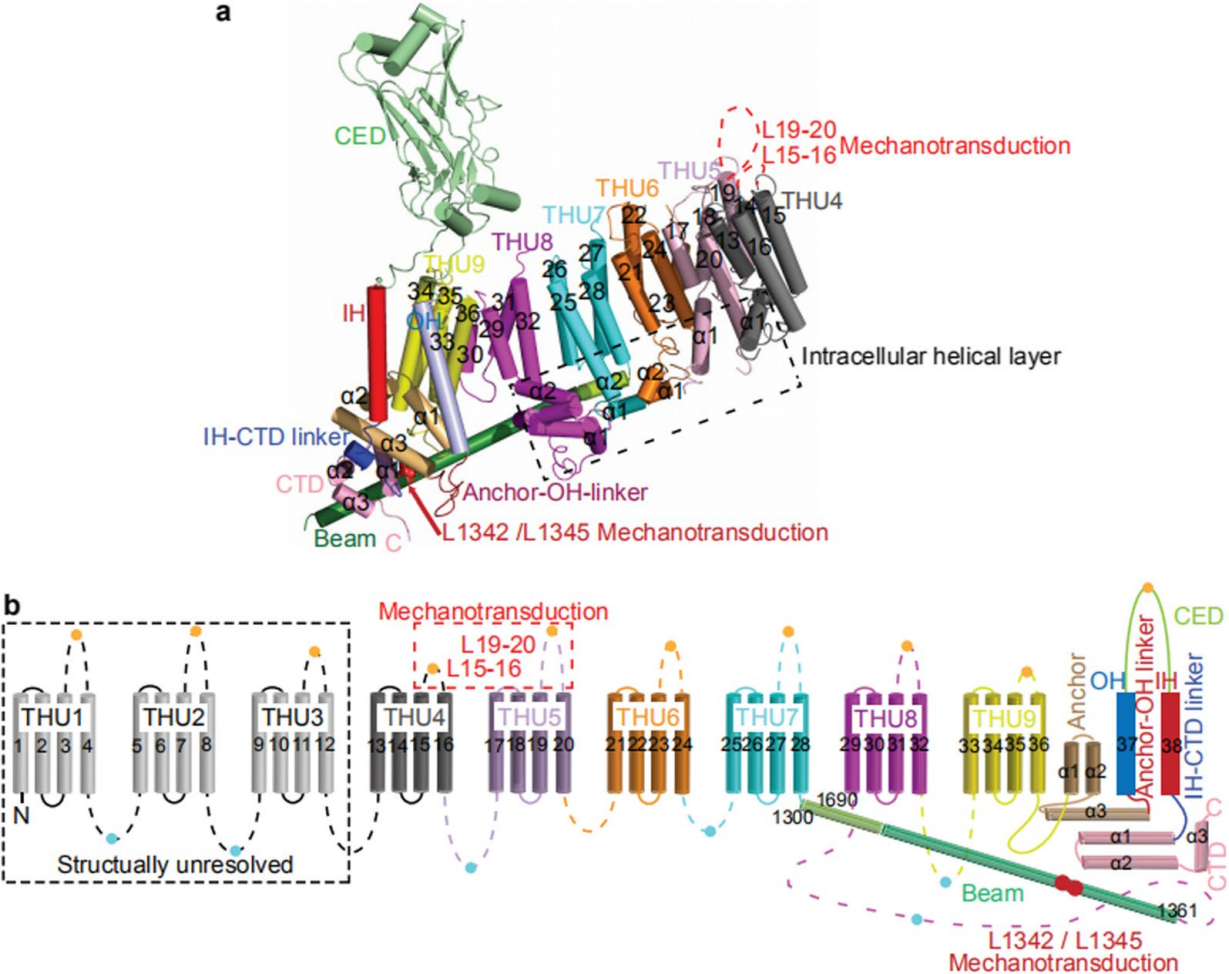
Structure of 38-TM topology model: **a** A cartoon model showing a cylindrical helix displayed a subunit with a single THU and major structural domains. **b** A 38-TM topology model for Piezo ion channels. (adapted from Zhao et al. [30])

In the past decade, a large number of studies elaborated on the molecular structure of Piezos. The structural determination of Piezo1 and Piezo2 using cryo-electron microscopy (cryo-EM) has greatly contributed to our understanding of the structure–function relationship of the complex Piezo channels [25, 26]. Ge Jingpeng et al*.* [27] reported the structure of mouse Piezo1 in 2015 for the first time. Using protein engineering, negative-staining electron microscopy, cryo-EM, and X-ray crystallography, they found that Piezo1 is a huge channel protein and has a three-bladed, propeller-like homotrimeric architecture with a central ion-conducting pore module topped with an extracellular cap domain structure containing 2547 amino acids. Subsequent studies resolved 26 out of the 38 TMs in each blade and central pore structure of Piezo1 and analyzed the gating mechanism of Piezos from the high-resolution results in conjunction with mutagenesis experiments that altered key amino acids [25, 28–30] (Fig. 1).

Mouse Piezo2, with 2822 residues, shares about 42% of its sequence with Piezo1, and both Piezo1 and Piezo2 have remarkable 38-TM topological organization. The 38-TM topological organization includes the N-terminal region, the C-terminal pore module, and the connecting beam and anchor domains. The N-terminal region was reported to be essential for mechanical force sensing [27, 31]. On the other hand, a study reported that several pathogenic and gain-of-function mutations are clustered in the C-terminal region. Chimeras between mouse Piezo1 and *Drosophila* Piezo, and Piezo1 and Piezo2 also verified that the intrinsic inactivation kinetics is primarily determined by the C-terminal pore region. Although Piezo1 and Piezo2 have similar structures, they play different roles in the respiratory system.

## Piezo1 in the respiratory system

### The expression of Piezo1 in the respiratory system

**Fig. 2:**
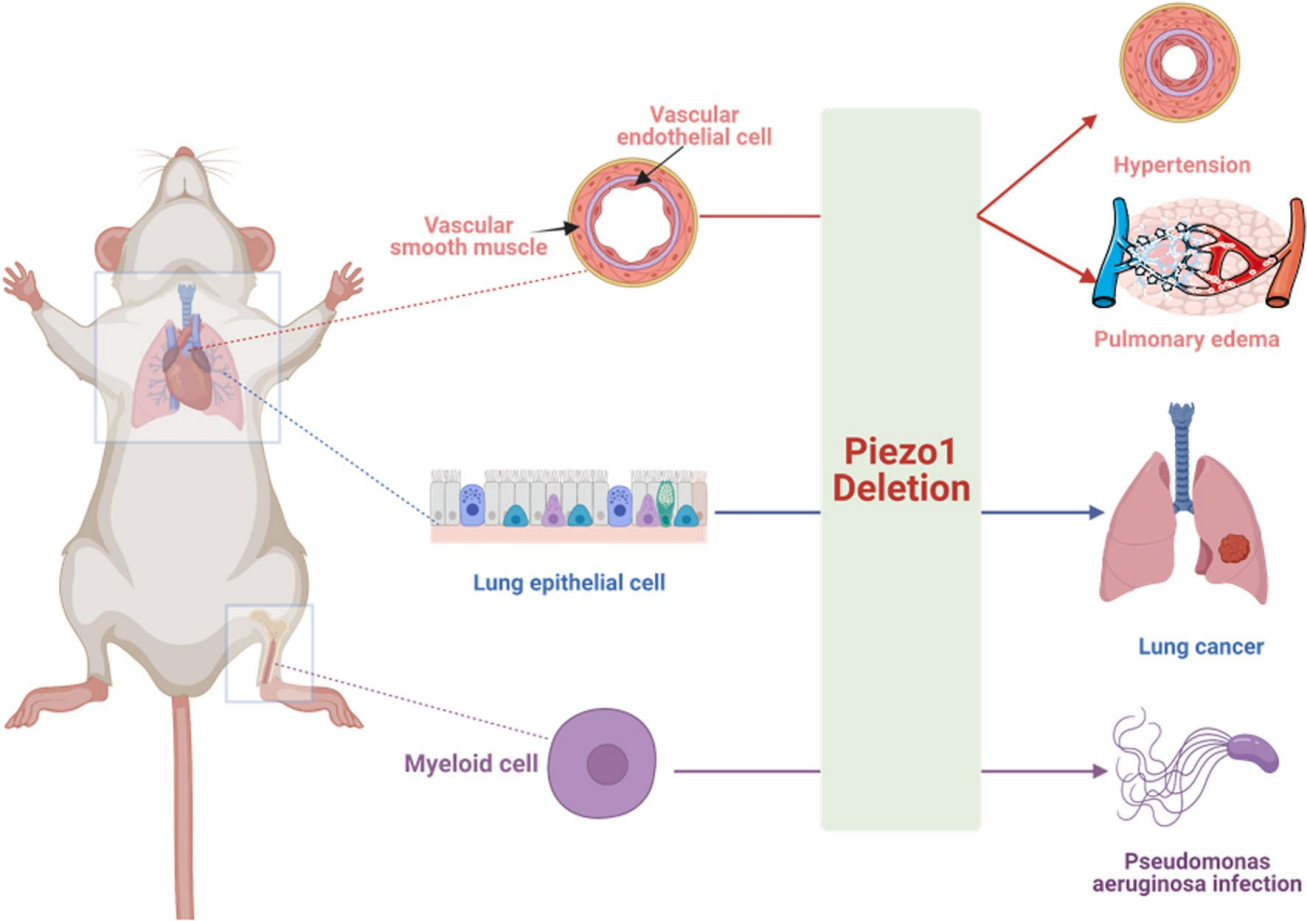
The expression of Piezo1 in respiratory system and problems might be caused by Piezo1 deletion (Created with BioRender.com)

Piezo1 ion channel is a determinant of vascular architecture during early development. It is readily expressed in a variety of ECs, especially in endothelium and endocardium, that are a part of the vascular system and can directly sense physiological shear stress in the vascular system [32]. The pulmonary circulation is a low-pressure vascular bed in which pulmonary ECs are subjected to mechanical forces during lung expansion [33]. Piezo1 is also expressed in pulmonary ECs (Fig. 2, Table 1). It was speculated that conditional knockout of endothelial Piezo1 in mice might result in death at E9.5-E11.5 of embryonic development [34]. Piezo1 is also highly expressed in smooth muscle cells (SMCs) of small-diameter arteries and is involved in arterial remodeling [18, 33] (Fig. 2, Table 1). Besides, smooth-muscle-specific Piezo1 knockout could impair the activity of stretch-activated ion channels [18]. Moreover, Piezo1 is expressed in lung epithelial cells to regulate cell division and crowding [35, 36]. It is possible that depletion of epithelial Piezo1 leads to a shift in an integrin-dependent pattern of anchorage independence and reduced cell migration/invasion [35]. Also, Piezo1 expressed in myeloid cells acts as a sensor of cyclical pressure [19] to resist the invading bacterial infection.

**Table 1:** Conditions and mechanisms caused by deletion of Piezo1 ion channel

Deletion of Piezo	Disease	Description	Mechanism
Global deletion	Died within 24 h after birth	NA	NA
Endothelial cells in embryo	Died at E9.5-E11.5	NA	Impaired endothelial cell alignment in response to shear stress; failing to remodel arteries
Endothelial cells in adult	Pulmonary edema	Increased pulmonary vascular hyperpermeability	Calpain in endothelial cleaved Src kinase to restore stability of the endothelial barrier
	PH	Pulmonary vascular remodeling	Failed to control the production of endothelial [Ca^2+^]i and NO
Smooth muscle cells	PH	Narrowed of the arterial diameter and thicken the arterial wall	Piezo1 has a trophic effect on resistance arteries, which could influence its diameter and wall thickness in hypertension
Epithelial cells	NSCLC	Worsen prognosis of patients	Piezo regulate cell migration and tumor growth
Myeloid cells	Lung inflammation	Caused Pseudomonas aeruginosa infection	Impaired expression of monocyte-dependent endothelin-1, stabilization of HIF1α

E9.5-E11.5: Embryo9.5-11.5; NA: not applicable; NSCLC: non-small cell lung cancer; PH: pulmonary hypertension

### Piezo1 in pulmonary hypertension

As a pulmonary vasodilator, Piezo1 inhibits pulmonary vasoconstriction [10, 37] and might play a key role in PH under hypoxic conditions [38]. The opening of the Piezo1 ion channel in SMCs has a trophic effect on resistant arteries, influencing their diameter and wall thickness in hypertension [18], which might due to the increased the influx of Ca^2+^ and activation of transglutaminase II. At the same time, Rode et al*.* [39] claimed that Piezo1 could elevate blood pressure during exercising while does not affect blood pressure during inactive. It is possible that increased blood flow in exercise increases Piezo1-mediated membrane depolarization, which in turn activates voltage-gated Ca^2+^ channels in adjacent vascular smooth muscle, leading to vasoconstriction.

On the other hand, a study showed that compared to normal participants, patients with intrapulmonary arterial hypertension exhibited significantly elevated Piezo1 levels in pulmonary artery endothelial cells (PAECs) (1.00 ± 0.09 vs 6.20 ± 1.78, n = 5, P < 0.01) [38]. Similarly, Wang et al*.* [40] demonstrated that Piezo1 was significantly upregulated in PAECs of patients and mice with idiopathic pulmonary hypertension. They reported that the level of Piezo1 in PAEC was higher in hypo-osmotic (200 mOsm/kg) solution. In a hypo-osmotic environment, Piezo1 in ECs could enhance the membrane stretch and Ca^2+^-dependent phosphorylation of AKT or ERK [41], and subsequently upregulates expression of Notch ligands (Jag-1/2, and DLL4) in PAECs [40]. Audrey Lhomme et al*.* used mouse and human PAECs to investigate the effect of Piezo1 channel activation on pulmonary vascular tone and found that intrapulmonary vascular relaxation could be promoted via Piezo1 channels by controlling endothelial [Ca^2+^]i and NO production [42]. However, they found that the magnitude of the depolarization of Yoda1 (Piezo1 ion channel activator) in human PAECs was much smaller than that reported by Rode et al. [39] for mesenteric artery depolarization (5 mV vs 20 mV). This difference may be related to the increased presence of Ca^2+^-activated potassium channels in PAECs, whose activation masks the depolarizing influx of sodium through Piezo1 channels [43].

### Piezo1 in pulmonary edema

Activation of Piezo1 in ECs induced by elevated pulmonary microvascular pressure mediates capillary ‘stress failure’ and pulmonary edema [20, 34]. Pulmonary edema is the result of disruption of the lung endothelial barrier, particularly the disruption of endothelial adhesion junctions (AJs) ^44,45^. Friedrich et al*.* [20] demonstrated that Piezo1 is associated with increased pulmonary microvascular pressure and endothelial barrier disruption. They conditionally knocked out Piezo1 in pulmonary ECs (Piezo1^iΔEC^) and elevated pulmonary microvascular pressure and found that Piezo1^iΔEC^ mice did not develop pulmonary endothelial barrier leakage and edema. Endothelial AJs proteins might exhibit a unique restrictive barrier in Piezo1^iΔEC^ mice. The authors suggested that Piezo1 signals pulmonary vascular hyperpermeability by promoting the internalization and degradation of endothelial AJs (calmodulin). The absence of Piezo1 or inhibition of calpain in ECs prevents the reduction in AJ proteins. Zhong et al*.* [46] demonstrated that in mechanically ventilated mice, the lung vascular permeability was significantly increased in Piezo1^iΔEC^ mice than in Piezo1^fl/fl^ mice, which could respond to high-volume mechanical ventilation. They showed that the downregulation of endothelial Piezo1 signaling might be an essential factor in the pathogenesis of ventilator-induced lung injury [46]. Influence may arise from the function of calpain in endothelial, which could cleave Src kinase to restore stability of the endothelial barrier [46].

### Piezo1 in lung cancer

Lung cancer is one of the most common fatal diseases in the world. Expression of Piezo1 in epithelial cells was also reported to influence the development of non-small cell lung cancer (NSCLC) [35]. Studies have analyzed the mRNA expression of Piezo1 in NSCLC and normal tissues and found that the expression was significantly lower in NSCLC compared to the normal tissues [35, 47]. Database with 1432 NSCLC patients reported that high mRNA expression of Piezo ion channel correlated with better overall survival of NSCLC patients [HR 0.79 (0.64–0.89)], especially in lung adenocarcinoma [HR 0.62 (0.46–0.84)] and female patients [HR 0.68 (0.51–0.89)] [47]. This may be due to the role of Piezo to regulate cell migration and tumor growth [35].

### Piezo1 in lung inflammation

Direct recognition of invading pathogens by innate immune cells is a key factor in the inflammatory response. Solis et al*.* demonstrated that Piezo1 expressed in myeloid cells might be associated with inflammation in the lung [19]. They reported that the knockdown of Piezo1 in myeloid cells showed diminished lung inflammation in the presence of bacterial infection in mice. A possible reason was that Piezo1 in myeloid cells could respond to cyclical hydrostatic pressure (CHP), which triggered the upregulation of proinflammatory genes such as IL1β and Cxcl10 via lipopolysaccharide (LPS). Moreover, mice with conditionally Piezo1-deficient myeloid cells (Piezo1^ΔLysM^) showed a marked susceptibility to *Pseudomonas aeruginosa* infection. The authors also found that despite the absence of the classical pattern recognition receptor signaling pathway, mechanical stimulation of macrophages and monocytes triggered an efficient and selective expression of pro-inflammatory and chemoattractant mediators in vitro. This inflammatory mechanosensory response is entirely dependent on Piezo1. It might result from impaired expression of monocyte-dependent endothelin-1, stabilization of HIF1α, and production of proinflammatory mediators.

Moreover, authors used bleomycin-induced pulmonary fibrosis model to investigate if Piezo1 in myeloid cell could drive autoinflammatory disease and found that there was lower level of lung damage in Piezo1^ΔLysM^ mice [19]. Piezo1 signaling in myeloid cells exacerbates pulmonary fibrosis, suggesting that mechanosensation in the altered microenvironment of fibrotic tissue can itself trigger auto-inflammation.

## Piezo2 in the respiratory system

### The expression of Piezo2 in extrapulmonary and intrapulmonary airways

**Fig. 3:**
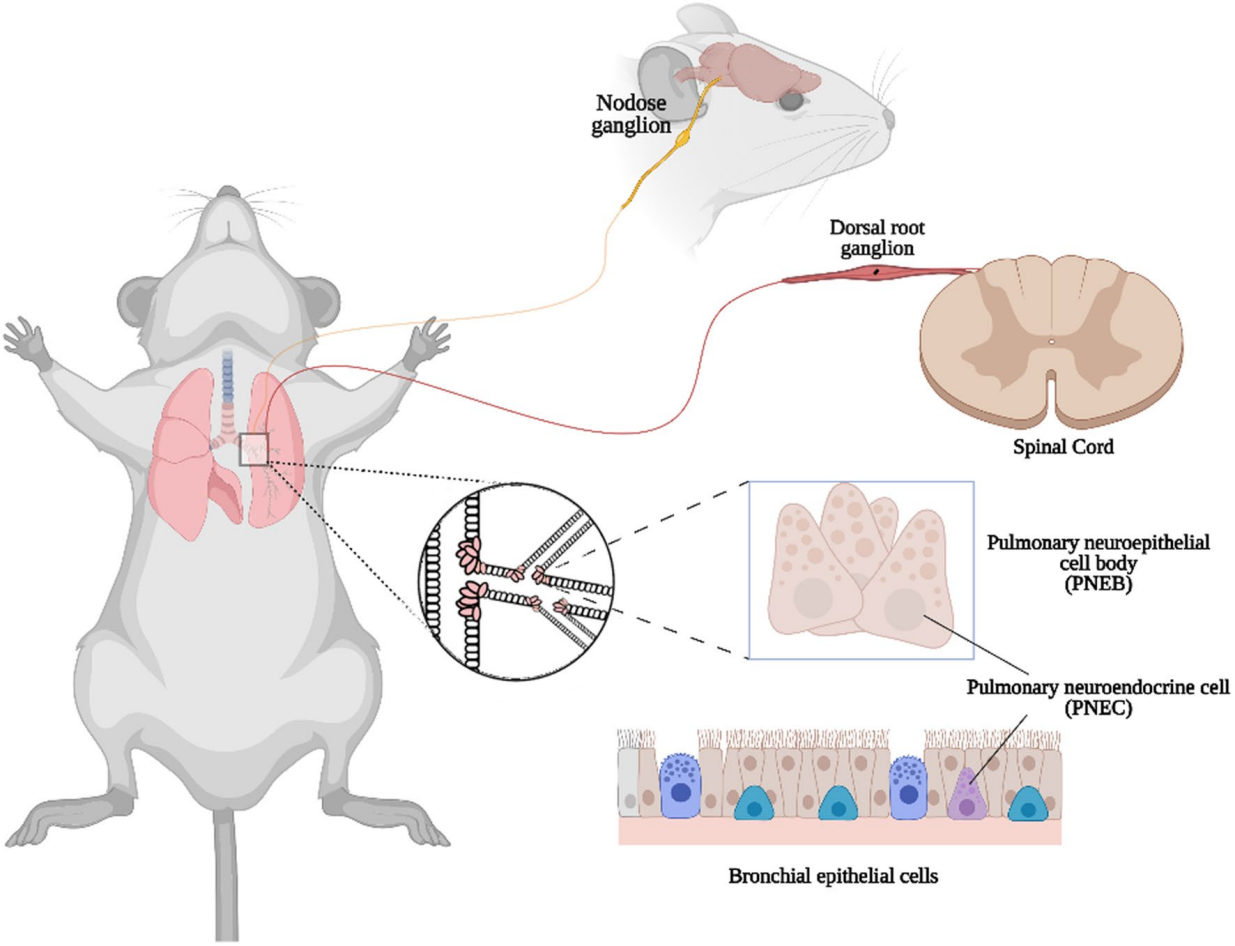
The expression of Piezo2 in respiratory system and neurons that correlated to respiration (Created with BioRender.com)

Piezo2 is an important mechanotransduction channel for a tactile response, proprioception, and mechanical pain in various types of neurons [48–50]. Unlike in the case of tactile response and proprioception, Piezo2 activity in sensory neurons that innervate the lung is critical for sensing organ stretch in adult mice [21]. Researchers used a lineage tracing method and detected GFP expression in the jugular-nodose ganglia complex, in which vagal cell bodies are located [21] (Fig. 3, Table 2). Sensory neuron-specific conditional knockout Piezo2 mice exhibit reduced adaptive neurons, mechanosensitive endings, and nerve endings impulse firing in response to mechanical stimuli [51].

**Table 2:** Conditions and mechanisms caused by deletion of Piezo2 ion channel

Deletion of Piezo	Disease	Description	Mechanism
Nodose ganglia neurons	Impaired Hering-Breuer reflex	Lung over-expansion	Piezo2 transduced the stretch of airway innervating vagal neurons
Jugular, trigeminal, and DRG neurons	Died within 24 h after birth	Respiratory distress and smaller airspaces	NA
NEB	ARDS	Increased fluid in the interstitial and/or alveolar space	Neuropeptide-induced barrier disruption
	Allergic asthma	Promoted smooth muscle contraction, eosinophil infiltration, and goblet cell hyperplasia	PNEC act through CGRP to stimulate ILC2s and elicit downstream immune responses
Hippocampus	OSAHS	Decreased the intracranial blood oxygen concentration and damaged hippocampal neurons	NA

CGRP: calcitonin gene-related peptide; ILC2: group 2 innate lymphoid cells; NEB: neuroepithelial body; NA: not applicable; OSAHS: obstructive sleep apnea hypopnea syndrome; PNEC: pulmonary neuroendocrine cells

A recent study was conducted to verify the distribution of Piezo2 in lung sections from healthy subjects and emphysema and lung fibrosis patients. They found that Piezo2 staining was localized to bronchial epithelial cells, macrophages, and smooth muscle cells [52]. Keiko et al*.* used the Piezo2^GFP^ reporter line to investigate where Piezo2 is expressed in the airway tract of mice and found that the GFP expression was detected in neuroepithelial body cells (NEBs) [21] (Fig. 3, Table 2).

### Role of Piezo2 in establishing respiration

Piezo2 could be critical for establishing newborn respiration. Mice with Piezo2 knockout in the jugular-nodose ganglia, trigeminal nerve, and DRG often die within 24 h after birth due to respiratory failure and inability to suckle [21, 53]. In humans, infants born with Piezo2 deficiency require emergency oxygen support at birth and continue to exhibit diminished respiratory support and shallow breathing throughout life [7, 54]. Delle et al*.* reported 10 patients with double allele deletion of Piezo2 who developed muscle atrophy with perinatal respiratory distress; however, all patients recovered respiratory function on their own within 24 h after birth [55]. These results suggested that Piezo2 is required for the transition from umbilical cord oxygen supply to respiration in neonatal mammals. Proper lung expansion and initiation of effective respiration in neonatal mice depended on the expression of Piezo2 in neural crest origin [21]. However, the stimuli that drive the firing of these neurons remain unknown, and the role of mechanical transduction in neonatal respiratory processes is currently unclear.

### Piezo2 channel activation by mechanical stimulation

During respiration, the lungs experience extensive mechanical forces, and these mechanical forces in the airway are thought to be cues that trigger physiological responses in the lungs [22]. The airway is innervated by both the vagus nerve and spinal sensory nerves [56, 57]. Vagal sensory neurons transmit basic sensory information (e.g., lung inflation [22, 56], arterial oxygen pressure, etc.) to the respiratory center in the brainstem. Studies showed that compared to rats with an intact vagal nerve, vagotomized rats had a 1.7-fold increase in tidal volume and a 2.4-fold decrease in the respiratory rate in the absence of appropriate vagal sensory feedback [56, 58]. Similarly, the conditional knockout of Piezo2 in vagal sensory neurons led to a 1.3-fold increase in the tidal volume. A recent study identified two distinct subtypes of vagal sensory neurons that innervate the lung and have unique functions: activation of neuron P2ry1, which expresses purinergic receptors and induces apnea, and the activation of neuron Npy2r, which expresses neuropeptide receptors and leads to rapid, shallow breathing [59].

Overactivation of Piezo2^+^ vagal neurons might overtly increase lung stretch responses in patients and lead to respiratory complications. A study reported that ablation of Piezo2 in sensory neurons of adult mice resulted in decreased neuronal response to lung inflation and impaired Hering-Breuer reflex [21]. They measured Hering-Breuer mechanoreflex in the conditional knockout of Piezo2 in nodose-ganglia complex and found that Piezo2-deficient mice had normal respiration as their lungs were inflated [21]. This indicated that Piezo2 expressed in the nodose-ganglia complex might be the major mechanotransducer required for the Hering-Breuer reflex. Moreover, abnormal activation of Piezo2 might be related to the occurrence of chronic pulmonary obstructive disease (COPD), in which the Hering-Breuer reflex is impaired [60].

### Piezo2 channel activation by chemical stimulation

Piezo2 expressed in NEBs might affect respiration. In the lungs, NEBs consist of clustered pulmonary neuroendocrine cells (PNECs), which represent a rare and evolutionarily conserved airway epithelial cell population [61]. The close association of Piezo2 with specific excitable NEBs is highly suggestive of a more complex signaling pathway. NEBs are organized in close association with a large number of nerve endings and are suitable to perceive changes in the airway environment and transmit this information to the central nervous system [61, 62]. They could be activated by a variety of stimuli and are thought to monitor all aspects of lung physiology, including inhalation, chemical, and mechanical changes [63]. According to the "chemical" hypothesis of mechanotransduction, NEBs should be able to respond to mechanical stimuli by secreting chemical signals and activating their respective molecular receptors on myelinated vagal sensory terminals [64, 65]. They could release bioactive substances, such as serotonin (5-hydroxytryptamine, 5-HT), substance P, and calcitonin gene-related peptide upon stimulation, and selectively engage mainly with vagal afferent nerve endings.

Increased NEBs and even increased PNEC products have been recognized in several respiratory diseases previously, such as bronchopulmonary dysplasia, COPD, ARDS [66], and allergic asthma [67]. There was a rare study that discussed the involvement of Piezo2, expressed in NEBs, in these diseases. On the other hand, the role of Piezo2 in NEBs might be similar to that in enterochromaffin cells [68]. Alcaino et al*.* showed that in visceral sensory neurons, Piezo2 is expressed in a subset of intestinal epithelial enteroendocrine cells and intestinal chromophores [17]. This subset of cells could generate a rapid inward ionic current that increases calcium ions in Piezo2-dependent intestinal epithelial enteroendocrine cells, while leading to a Piezo2-dependent mechanical sensitive 5-HT release, and regulating intestinal fluid secretion.

### Respiratory role of Piezo2 expressed in brain tissues

Breathing does not only help transport oxygen to the lungs to keep us alive, it also affects how we think and feel. During breathing, significant changes are observed in the activity of the hippocampus and amygdala in the brain. Christina Zelano et al*.* reported that when someone inhales, neurons in the olfactory cortex, amygdala, and hippocampus, all of which travel through the brain's limbic system, are stimulated [69]. They claimed that the memory of people might be better during inhalation than exhalation. Obstructive sleep apnea/ hypopnea syndrome (OSAHS) leads to a decrease in intracranial blood oxygen concentration and damages hippocampal neurons. Piezo2 expressed in the hippocampus might explain the relationship between the brain and the respiratory system [70]. However, existing evidence does not prove that OSAHS is related to the expression of Piezo2 in the hippocampus.

## Conclusion and future perspective

In conclusion, Piezo ion channels play a mechanosensitive and mechanotransductive role in lung diseases. Alveolar epithelial and endothelial cells experience mechanical forces during respiration. Deficiency of Piezo1 or mutation in Piezo2 leads to impaired epithelial cell adhesion and increased cell migration [35], which may promote tissue repair in ARDS and NSCLC. Although this issue has not been investigated, the alveolar epithelial expression of Piezo1 and Piezo2 may regulate the response to distraction-induced or ventilation-induced lung injury. Therefore, Piezo ion channels may play an important role in multiple aspects of lung biology.

Although our understanding of the mechanisms of mechanotransduction has advanced since the discovery of Piezo channels, several fundamental questions regarding the regulatory role of Piezo channels in the lung remain unanswered. For example, research verifying the mechanism underlying the effect of Piezo2 on the Hering-Breuer reflex is lacking. Similarly, research on Piezos in the pulmonary epithelium and endothelium will yield important insights into lung physiology and pathophysiology.

## Supplementary Information

**Additional file 1. **Expression of Piezo ion channels in human tissues.

## Data Availability

Not applicable.

## Abbreviations

5-HT: 5-Hydroxytryptamine
AJ: Adhesion junction
CHP: Cyclical hydrostatic pressure
cryo-EM: Cryo-electron microscopy
COPD: Chronic pulmonary obstructive disease
DRG: Dorsal root ganglia
ECs: Endothelial cells
LPS: Lipopolysaccharide
MA: Mechanically activated
NSCLC: Non-small cell lung cancer
OSAHS: Obstructive sleep apnea/hypopnea syndrome
PAECs: Pulmonary artery endothelial cells
PH: Pulmonary hypertension
PNECs: Pulmonary neuroendocrine cells
TMs: Transmembrane domains
