# Supplementary material for: Mechanosensitive Piezo channels mediate the physiological and pathophysiological changes in the respiratory system
Source: Respir Res. 2022 Jul 29;23:196. doi: 10.1186/s12931-022-02122-6 (PMC9338466; doi:10.1186/s12931-022-02122-6)
Supplement: Supplementary file 1 — Additional file 1. Expression of Piezo ion channels in human tissues. [file 12931_2022_2122_MOESM1_ESM.docx]

**Supplementary**


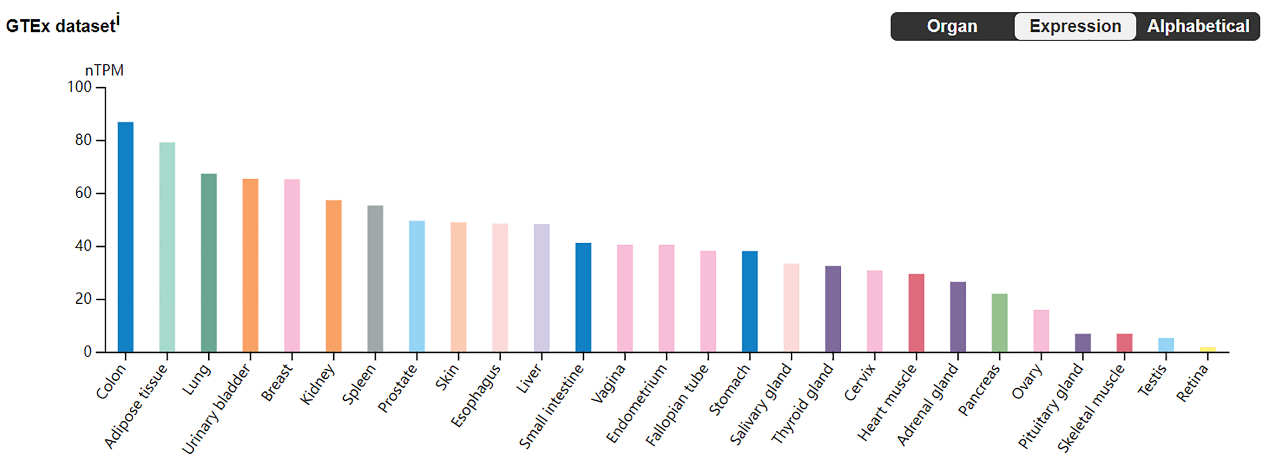


Figure1. Piezo1 ion channel expressed in various tissues of human (Atlas database).


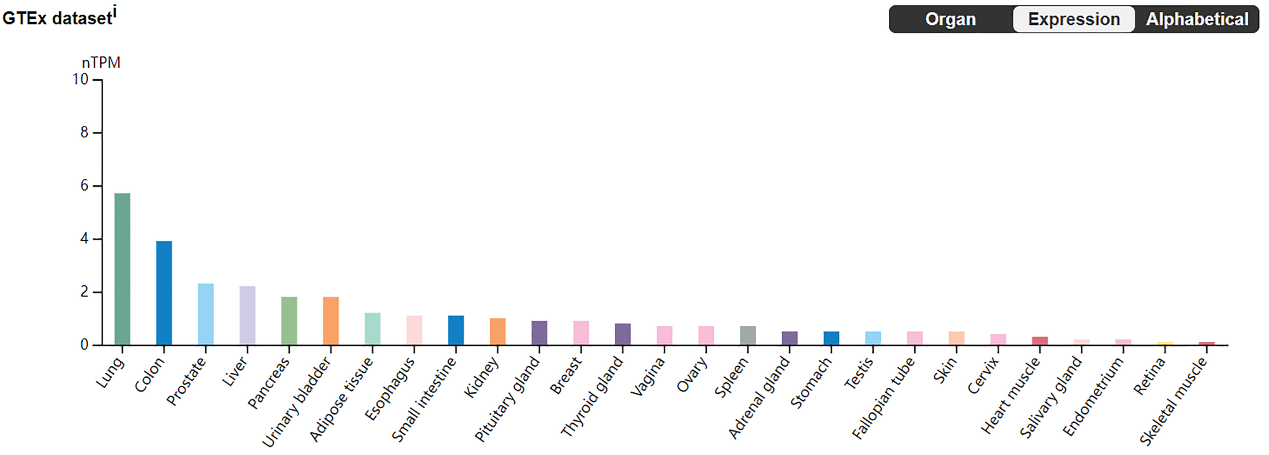


Figure2. Piezo2 ion channel expressed in various tissues of human (Atlas database)
